# Fracture and plastering of distal left main stent during double-kissing Culotte technique: a case report

**DOI:** 10.1093/ehjcr/ytae215

**Published:** 2024-04-24

**Authors:** Saibal Mukhopadhyay, Ghazi Muheeb, Jamal Yusuf, Sanjeev Kathuria

**Affiliations:** Department of Cardiology, Govind Ballabh Pant Institute of Post Graduate Medical Education and Research, Jawaharlal Nehru Marg, New Delhi, Delhi 110002, India; Department of Cardiology, Govind Ballabh Pant Institute of Post Graduate Medical Education and Research, Jawaharlal Nehru Marg, New Delhi, Delhi 110002, India; Department of Cardiology, Govind Ballabh Pant Institute of Post Graduate Medical Education and Research, Jawaharlal Nehru Marg, New Delhi, Delhi 110002, India; Department of Cardiology, Govind Ballabh Pant Institute of Post Graduate Medical Education and Research, Jawaharlal Nehru Marg, New Delhi, Delhi 110002, India

**Keywords:** Left main, Bifurcation lesion, Double-kissing Culotte, Stent fracture, Optical coherence tomography, Case report

## Abstract

**Background:**

Acute fracture of a left main (LM) stent during angioplasty is a rare complication. Cardiologists should be aware of the risk of stent fracture (SF) following kissing balloon inflation (KBI) even if the effective diameter of the balloons does not exceed the recommended expansion limits of stents.

**Case summary:**

A 64-year-old female with hypertension and dyslipidaemia presented with crescendo angina since three months in spite of optimal medical therapy. Coronary angiogram showed a distal LM bifurcation lesion. The patient was admitted for LM bifurcation stenting by upfront two-stent technique (inverted double-kissing Culotte technique). Following first KBI of the stent placed from left circumflex artery (LCX) to LM, there was stent deformation in the LM shaft. As we had planned the Culotte technique, we decided to exclude the fractured segment by stenting from left anterior descending artery (LAD) to LM. The stent from LAD–LM successfully excluded the fractured part of the first stent from the lumen of LM. Optical coherence tomography done after final KBI from LCX–LM revealed successful exclusion of the deformed segment of the LCX stent with mild malapposition at the site of the deformed stent. A follow-up angiogram after six months showed normal in-stent flow with no evidence of restenosis or pseudoaneurysm.

**Discussion:**

Acute LM SF during coronary intervention can occur even if the effective cumulative diameter of the inflated balloons does not exceed the mentioned expansion limit of stents. Intravascular imaging is a helpful modality to define type of SF and its management.

**Learning points**

- Recommended *in vitro* stent expansion limits of various drug eluting stents may not translate clinically.
- During kissing balloon inflation, complication of stent fracture should always be kept in mind.
- In addition to fluoroscopy, optimal coherence tomography imaging is a useful modality to assess fracture and outcome following percutaneous management.

## Introduction

Acute fracture of a coronary stent during angioplasty is a dreadful complication. In the literature, the incidence of stent fracture (SF) varies from <1% to 20% depending on the time after implantation, stent type and, most importantly, the methods and definitions used for the diagnosis of SF.^1^ However, with increase in the number of stent implantations, an increasing number of SF cases are being reported, particularly following drug eluting stent implantation.^2^ Among all the SF, left main (LM) SF is the rarest.^3^

We report a case of acute LM SF during stenting by the DK (double-kissing) Culotte technique and its probable mechanism.

## Summary figure

Line diagram showing exclusion of the fractured segment (yellow) of the LCX stent (red) from the lumen of the LM by placing a second stent (blue) from LAD–LM through the struts of the LCX stent.

**Figure:**
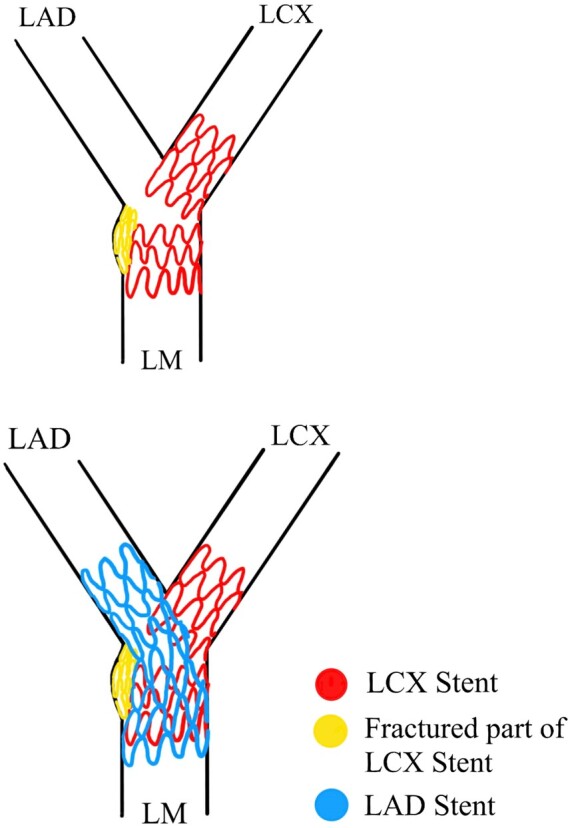


## Case presentation

**Figure 1:**
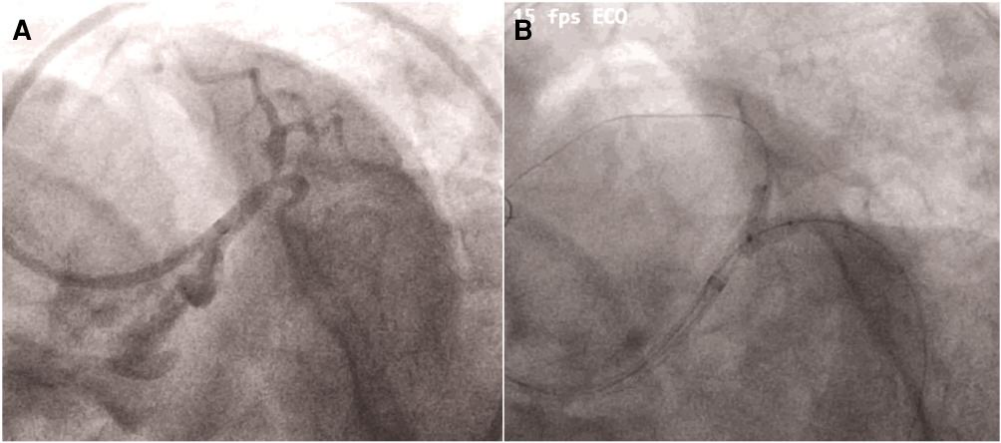
(*A*) CAG showing LM bifurcation lesion (Medina 1-1-1 and bifurcation angle of 45°). (*B*) First sequential KBI done with 3.5 × 10 NC balloon in LCX and a 3 × 12 mm NC balloon in LAD at 14 atm pressure.

A 64-year-old female with hypertension and dyslipidaemia had undergone coronary angiogram (CAG) one week ago at a peripheral centre for crescendo angina since three months in spite of being on optimal medical therapy (aspirin 75 mg o.d., clopidogrel 75 mg o.d., atorvastatin 40 mg h.s., metoprolol succinate extended release 100 mg o.d., telmisartan 40 mg o.d., and nitro-glycerine 6.5 mg b.i.d.). Her CAG revealed distal LM bifurcation lesion (Medina 1-1-1, angle of bifurcation ∼45°) with right dominant circulation and SYNTAX Score of 23 (*Figure 1A*, Supplementary material online, *Video S1*). Application of SYNTAX Score 2020 showed comparable 5 years major adverse cardiovascular events and 10 years mortality for coronary artery bypass surgery and percutaneous coronary intervention. Baseline echocardiography was normal with left ventricular ejection fraction of 60%. As patient opted for angioplasty, she was admitted for distal LM bifurcation stenting by an upfront two-stent strategy using the inverted DK mini-Culotte technique.^4,5^

**Figure 2:**
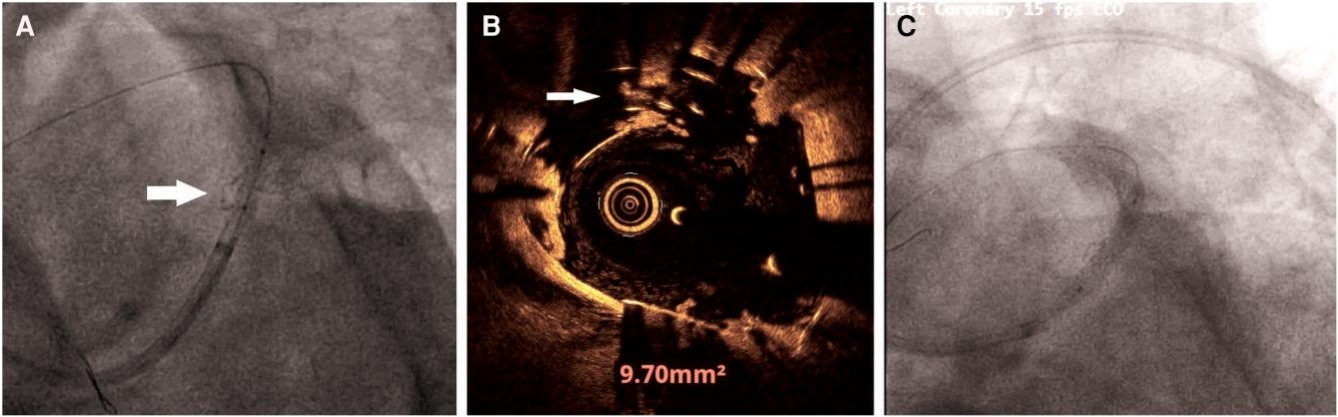
(*A*) CAG showing stent deformation in the LM shaft (arrow). (*B*) OCT image showing SF in the LM shaft (arrow). (*C*) LAD–LM stented with a 3.5 × 18 mm sirolimus eluting stent (Ultimaster™) excluding the deformed part of the previous stent from the LM lumen.

**Figure 3:**
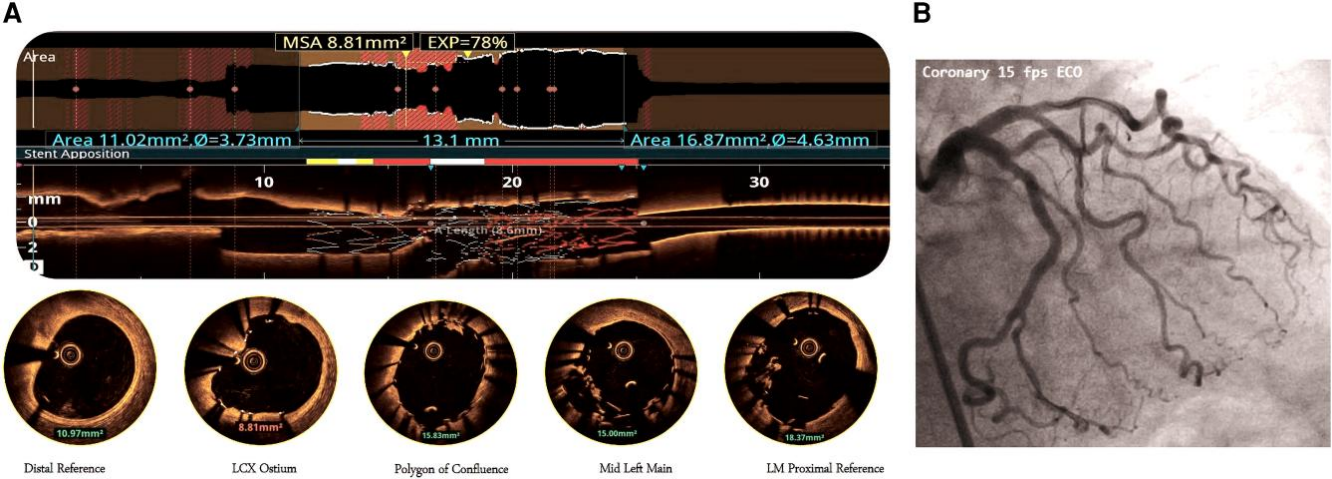
(*A*) OCT run from LCX showing good apposition of stent in the LCX, a proximal LCX area of 8.81 mm^2^, POC area of 15.83 mm^2^, and mild malapposition in the distal LM but good luminal area of 15.26 mm^2^. The deformed portion of the LCX stent can be seen plastered against the wall by the stent deployed from LAD–LM. (*B*) Final CAG showing TIMI-III flow in the left coronary system.

Right transfemoral route was used for the procedure. The LM was engaged with 7 F EBU 3.5 guide catheter (Launcher™, Medtronic, CA, USA). Following this, both, left anterior descending artery (LAD) and left circumflex artery (LCX), were wired with workhorse wires (ChoICE™ Floppy, Boston Scientific, Natick, MA, USA). We had done a baseline optical coherence tomography (OCT) to define the lesion length, morphology and stent size. Vessel diameters on OCT imaging of normal segments for the landing zone of stents were as follows: proximal LM reference external elastic lamina (EEL)-EEL diameter—4.6 mm, LAD reference EEL-EEL diameter of normal segment beyond lesion—3.73 mm, and LCX reference EEL-EEL diameter of normal segment beyond lesion—3.98 mm, respectively (see supplementary material online, *Figures S1* and *S2*, supplementary material online, *Videos S2* and *S3*). Pre-dilatation of ostio-proximal LAD and LCX was done with a cutting balloon 2.75 × 12 mm (Wolverine™, Boston Scientific, Natick, MA, USA) at 10–12 atm pressure. Following lesion preparation, LCX–LM stenting was done with a 3.5 × 15 mm sirolimus eluting stent (Ultimaster™, Terumo Corporation, Shibuyaku, Tokyo, Japan) deployed at 14 atm pressure. Following this, proximal optimization (POT) of the stent in LM was done with a 4 × 08 mm non-compliant (NC) balloon (NC Solarice™ RX, Medtronic, CA, USA) at 12 atm pressure. This was followed by a sequential kissing balloon inflation (KBI) with a 3.5 × 10 mm NC balloon first inflated up to its nominal pressure of 14 atm in the LCX (to fix the struts of the stent at the LCX ostium). Keeping this balloon inflated, another NC balloon of 3 × 12 mm (NC Solarice™ RX) size passed across the LM through the distal strut of the LCX stent towards LAD was inflated at nominal pressure of 14 atm (*Figure 1B*, supplementary material online, *Video S4*). Following sequential KBI, we noticed stent deformation at the LM shaft that was readily detectable on fluoroscopy (*Figure 2A*). Optical coherence tomography imaging from LCX–LM confirmed the SF (*Figure 2B*). As we had already planned the Culotte technique, we decided to promptly exclude the deformed segment from the intravascular lumen by placing a stent across it from LM–LAD. A 3.5 × 18 mm sirolimus eluting stent (Ultimaster™) was deployed from proximal LM–LAD excluding the deformed part of the previous stent from the LM lumen (*Figure 2C*, Summary figure). Proximal optimization of the proximal part of the stent in LM was done with 4.5 × 08 mm NC balloon (NC Solarice™ RX) at 14 atm pressure. After recrossing into LCX through the distal strut of the stent placed from LM–LAD, post-dilatation of the LAD stent was done with a 3.5 × 08 mm balloon (NC Solarice™ RX) at 18 atm pressure. Final KBI was done in the following manner. A 3.25 × 10 mm (NC Solarice™ RX) balloon was placed in LAD and 3.5 × 08 mm balloon (NC Solarice™ RX) through the struts of LAD stent towards LCX. First, the 3.25 mm LAD balloon was inflated at nominal pressure of 15 atm and keeping this balloon inflated it was followed by inflation of LCX balloon also at nominal pressure of 15 atm (to effectively open up the struts of the LAD stent towards LCX ostium) followed by simultaneous deflation of both the balloons (by Finet’s law,^6^ effective diameter of the 3.5 mm + 3.25 mm balloon was 4.45 mm) (see supplementary material online, *Video S5*). Following rePOT of LM with a 4.5 × 08 mm balloon, an OCT run was done. OCT run from LCX showed that the fractured segment was excluded with maintained integrity of the remaining stent and mild malapposition at the site of the fractured segment (see supplementary material online, *Videos S6* and *S7*). Final luminal areas achieved as assessed by OCT imaging were as follows: proximal LAD area of 6.90 mm^2^, proximal LCX area of 8.81 mm^2^, POC area of 15.83 mm^2^, and proximal LM luminal area of 15.26 mm^2^ (*Figure 3A*). The fractured segment of the LCX stent was plastered against the wall by the stent deployed from LAD–LM. Final CAG also showed TIMI-III flow in LAD and LCX (*Figure 3B*, supplementary material online, *Video S8*). Following stenting, as good luminal areas were achieved with no evidence of gross malapposition, we decided to keep the patient on dual antiplatelet therapy (DAPT) (aspirin 75 mg + clopidogrel 75 mg o.d.) plus low molecular weight heparin 60 mg subcutaneous b.i.d. for 72 h followed by DAPT alone that was to be continued for 12 months. She was observed for one week in the hospital before discharge though could have been discharged earlier (48 h after withdrawing low molecular weight heparin).

**Figure 4:**
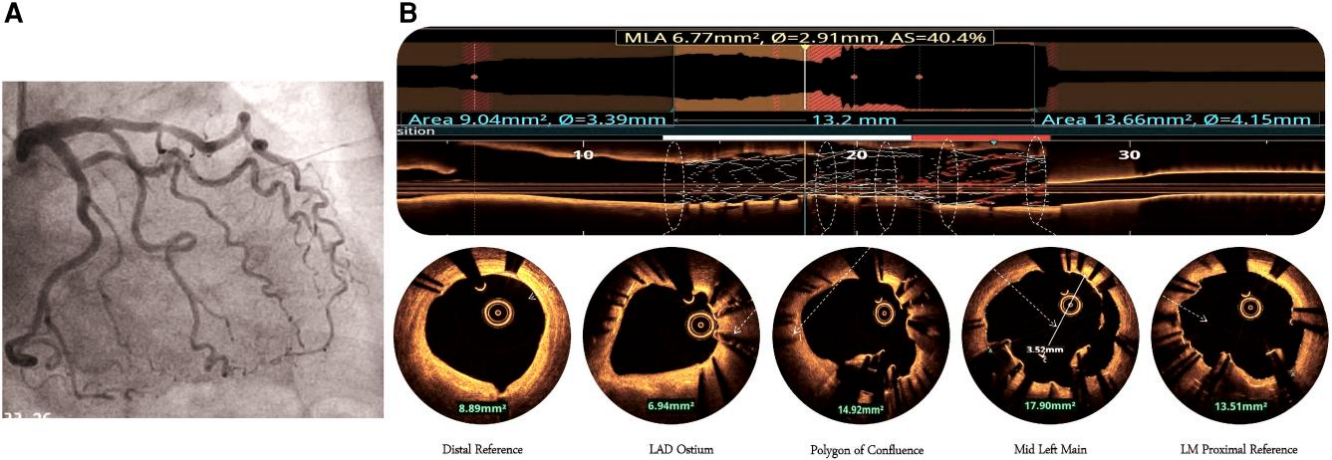
(*A*) Follow-up CAG showing normal in-stent flow and no evidence of any aneurysm formation of the deformed stent. (*B*) Follow-up OCT images from LAD–LM showing good endothelial healing with preserved luminal areas.

In view of atypical symptoms, a repeat CAG was undertaken after six months that showed normal in-stent flow and no evidence of any pseudoaneurysm formation at the site of deformed stent (*Figure 4A*). Repeat OCT imaging showed good endothelial healing with preserved luminal areas (*Figure 4B*).

## Discussion

The suggested predisposing factors associated with acute SF are overexpansion of stent by oversized balloon resulting in weakening of the struts, inappropriate handling of the stent, use of crush technique for bifurcation stenting, or deployment of stent in highly tortuous and angulated vessel.^7,8,9^ Stent fracture of the LM is very rare and is probably caused by rich elasticity of the LM.^10^

However, in our case, we feel that the factor responsible for SF was deployment of the stent in an angulated segment from LCX–LM that may have resulted in a non-uniform distribution of stress during KBI. The NC balloons used for first KBI were 3 × 12 mm in the LAD and 3.5 × 10 mm in the LCX. The balloon in the LCX was first inflated at 14 atm pressure to achieve a balloon diameter of 3.5 mm that was necessary to fix the 3.5 mm stent against the ostium and at this point the balloon placed in LAD across the distal strut of the LCX stent was inflated up to 14 atm pressure to dilate the stent strut followed by simultaneous deflation of both the balloons. As per the Finet’s law,^6^ the effective diameter of the two balloons in the LM was 0.67(3.5 + 3.0) = 4.35 mm. The expansion limit of a 3.5 mm Ultimaster™ stent has been stated to be 4.5 mm.^10^ Yet the stent got deformed and fractured at an effective diameter of 4.4 mm. This shows that the expansion limit of stents mentioned on the basis of *in vitro* studies may not translate *in vivo* particularly if deployed in angulated arteries and during KBI as there is a non-uniform and non-circular dilatation of the stent. Of the stents whose expansion limits are available in the literature^11^ [XIENCE Sierra™ (Abbott Vascular, Santa Clara, CA, USA), Resolute Onyx™ (Medtronic, CA, USA), Synergy™ (Boston Scientific, Natick, MA, USA), Ultimaster™ (Terumo Corporation, Shibuyaku, Tokyo, Japan), Orsiro™ (BIOTRONIK AG, Berlin, Germany)], we had only Ultimaster™ stent that was readily available on the shelf and hence it was used.

To preserve the architecture of the stent and prevent migration of stent struts, Toth *et al*.^4^ have recommended sequential KBI. The first sequential KBI was performed with a 3.0 × 12 mm NC balloon in the LAD and 3.5 × 10 mm NC balloon in the LCX. If based on OCT findings, we had used 3.5 mm balloons for first the KBI, the effective diameter of the two balloons during kissing would have been 4.69 mm as per the Finet’s Law^6^ [0.67 (3.5 + 3.5)], exceeding the expansion limit of a 3.5 mm Ultimaster™ stent (expansion limit is 4.5 mm).^11^ Hence, we decided to use a 3.5 mm balloon across the LCX and a 3 mm balloon across the struts of the stent towards the LAD [effective diameter of 4.35 mm as per the Finet’s law,^5^ 0.67(3.5 + 3.0)]. The balloons were sequentially inflated at 14 atm pressure, first in the LCX and then in the LAD followed by simultaneous deflation of both the balloons. But following the first KBI, we observed fracture of the shaft of the stent in LM in spite of the effective diameter of the inflated balloons not exceeding the expansion limit of the stent. The second KBI was also performed in a similar manner. A 3.25 × 10 mm NC balloon in LAD and 3.5 × 08 mm NC balloon in LCX was used so that the effective diameter of both the balloons in the LM does not exceed 4.5 mm as per the Finet’s law^6^ (see supplementary material online, *Videos S4* and *S5*).

In distal LM bifurcation upfront two-stent strategy, DK Crush was compared with Culotte stenting in DK CRUSH III trial and the main reason of the superiority of DK Crush over Culotte stenting was an increased incidence of repeat revascularization of LCX in the Culotte group due to increased incidence of ostial restenosis of the LCX.^12^ Chen *et al*.^12^ mentioned that the main reason of ostial restenosis at the LCX ostium was the potential deformity produced in the LCX ostium during the dilatation of struts of the LCX stent towards the LAD ostium during reverse Culotte (migration of stent struts from the ostial LCX towards LAD producing a potential gap at the LCX ostium resulting in an increased incidence of LCX ostial restenosis and target vessel revascularization). Toth *et al*.^4^ have demonstrated that after deployment of the first stent from side branch to the main vessel, dilatation of struts of the stent towards the distal main vessel for subsequent placement of stent results in migration of struts from the ostium of the side branch towards the distal main vessel leaving a potential gap in the side branch ostium that is a cause of subsequent restenosis in the side branch ostium. However, inflating a balloon from side branch to the main vessel prior to dilating the struts of the stent towards the distal main vessel with a balloon (sequential dilatation) and simultaneous deflation prevents stent struts at the side branch ostium from migrating towards the distal main vessel and development of a gap. They proposed that this additional kissing may prevent deformity of the stent at the side branch ostium and risk of restenosis. Hence, we decided to do inverted mini-DK Culotte in place of a conventional Culotte stenting as has also been recommended in the European Bifurcation Club 16th Consensus document.^13^

DK Crush is technically more challenging because:

- it requires crossing of stent struts non-distally prior to first kissing,
- it results in disruption of stent architecture due to crushing, and
- it creates three layers of stent in the proximal main vessel.

In the EBC Main trial, majority of the patients (53%) in upfront two-stent strategy underwent Culotte stenting and we felt that adoption of DK Culotte (resulting in maintained structural integrity of both side branch and main branch stents) will provide results equivalent to DK Crush and avoiding the limitations of DK Crush technique as mentioned above.^14^

Fluoroscopy alone or stent boost is useful to detect SF. In our case, fluoroscopy *per se* showed the deformity of stent in the wall opposite the LCX ostium following first KBI suggestive of type III SF as proposed by Nakazawa *et al*.^15^ (*Figure 2A*). As we had planned a Culotte technique, we immediately decided to exclude the deformed segment of the stent from the lumen of the LM by deploying a stent from LM–LAD through the distal struts of the deformed LCX stent. Following deployment of stent from LM–LAD and final KBI, we undertook an OCT run that showed that the deformed portion of the stent was plastered against the arterial wall by the second deployed stent. On the basis of OCT, this fracture was classified as type III as proposed by Gori *et al*.^16^ (*Figure 2B*). The added advantage of 3D reconstruction also helped us to confirm the structural integrity of the second stent and persistent localized fracture of the first stent following the final KBI (see supplementary material online, *Videos S6* and *S7*).

## Conclusion

This case highlights the need for interventional cardiologists to be aware of the risk of SF following KBI even if the effective diameter of the balloons does not exceed the recommended expansion limits of the stents. *In vitro* studies on stent expansion limits may not translate clinically particularly during KBI and can produce SF. Intravascular imaging is an important modality not only to plan LM interventions but also to detect complications and document its effective management.

## Lead author biography


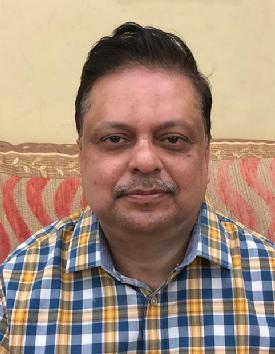


Dr Saibal Mukhopadhyay completed his post-graduation in Cardiology in 2002 from the University of Delhi (India). He joined the Department of Cardiology in Gobind Ballabh Pant Hospital as senior research associate and then as faculty in the year 2005. He is currently serving as the Head of Department in this institute. Several of his research articles in the field of cardiology have been published in national and international journals.

## Supplementary Material

ytae215_Supplementary_Data

## Data Availability

The data underlying this article are available in the article and in its online Supplementary material.
