# Supplementary figures and images for: Fracture and plastering of distal left main stent during double-kissing Culotte technique: a case report
Source: Eur Heart J Case Rep. 2024 Apr 24;8(5):ytae215. doi: 10.1093/ehjcr/ytae215 (PMC11098036; doi:10.1093/ehjcr/ytae215)

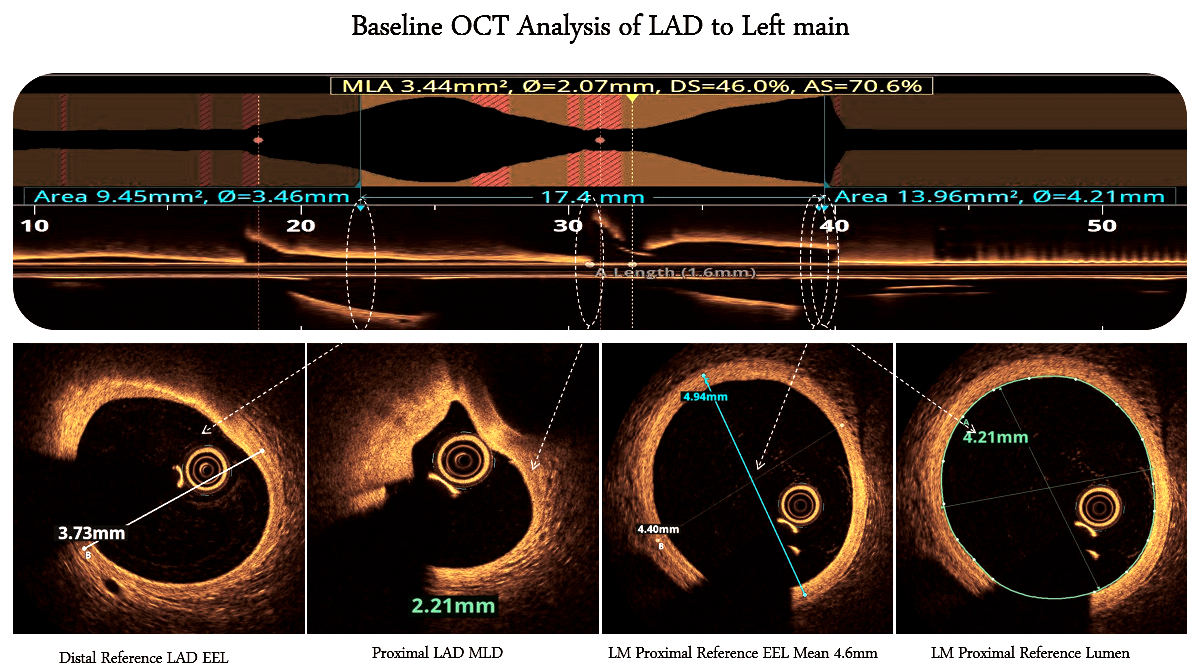

Supplement: ytae215_Supplementary_Data [file ytae215_supplementary_data.zip › Suppl._figure_1_cmyk300.tiff]

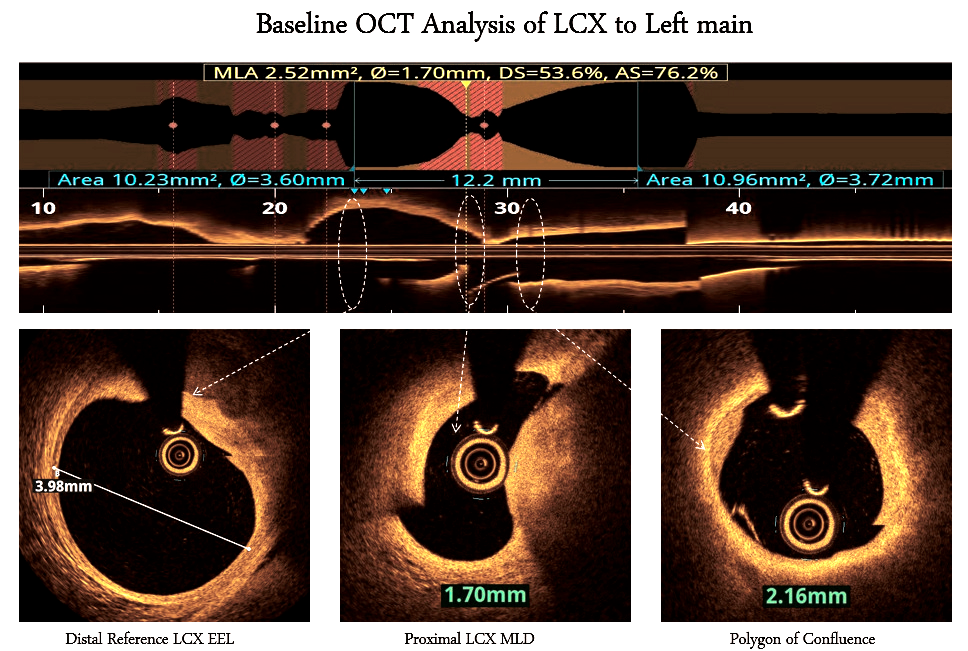

Supplement: ytae215_Supplementary_Data [file ytae215_supplementary_data.zip › Suppl._figure_2_cmyk300.tiff]
